# An animal model manifesting neurodegeneration and obesity

**DOI:** 10.18632/aging.100172

**Published:** 2010-07-08

**Authors:** Etsuo Susaki, Keiichi I. Nakayama

**Affiliations:** ^1^ Department of Molecular and Cellular Biology, Medical Institute of Bioregulation, Kyushu University, 3-1-1 Maidashi, Higashi-ku, Fukuoka, Fukuoka 812-8582, Japan; ^2^ CREST, Japan Science and Technology Agency, 4-1-8 Honcho, Kawaguchi, Saitama 332-0012, Japan

**Keywords:** Ubiquitin, mouse model, neurodegeneration, obesity

## Abstract

Although the existence of a link between neurodegenerative diseases and obesity has been suggested, a causal relation between neural degeneration and obesity has remained to be demonstrated experimentally. We recently showed that neurodegeneration in the hypothalamic satiety center results in obesity in mice transgenic for E4B (also known as UFD2a), a mammalian ubiquitin elongation factor (E4). Increased expression of E4B in neurons of the transgenic mice results in the formation of ubiquitin-positive aggregates similar to those apparent in many human neurodegenerative diseases as well as in degeneration of hypothalamic neurons responsible for the regulation of food intake and energy expenditure. We thus propose that neurodegeneration is a possible cause of human obesity and related metabolic diseases, which have become a serious public health problem worldwide. Our animal model is thus a powerful tool for studies of the relation between neurodegeneration and obesity.

Aging of the human population is a key concern worldwide because of the associated social and medical problems. Important diseases related to aging include neurodegenerative conditions, such as Alzheimer's disease, most of which are characterized by the formation of intracellular protein aggregates in neurons and neuronal loss. Individuals with such diseases exhibit various neural disorders including motor, cognitive, and behavioral dysfunction. Another disease that has traditionally been associated with aging is obesity, although this condition, together with its accompanying metabolic abnormalities, has recently also begun to affect younger individuals as a result of changes in diet and lifestyle and has become a serious public health problem worldwide. A link between these two types of disease has been postulated on the basis of their association with aging. Indeed, the possible relation between neurodegeneration and obesity in animal models or humans has been studied now for several decades. However, most such studies have focused on the possibility that obesity and related metabolic disorders exacerbate neurodegeneration and thereby promote cognitive decline and increase vulnerability to brain injury [1]. Few studies have addressed the possibility that neurodegeneration in the brain may cause obesity, as is suggested by the identification of hereditary neurodegenerative disorders associated with obesity such as Prader-Willi syndrome [2].

## E4 as a new player in the ubiquitin-proteasome system

A key focus of our research group has been the functions and underlying mechanisms of the ubiquitin-proteasome system (UPS). The UPS plays an important role in the elimination of short-lived regulatory proteins [3], including those that contribute to such processes as the cell cycle, cellular signaling in response to environmental stress or extracellular ligands, morphogenesis, secretion, DNA repair, and organelle biogenesis [3-5]. The UPS pathway includes two key steps: covalent attachment of multiple ubiquitin molecules to the protein substrate and degradation of the ubiquitylated protein by the 26S proteasome complex. The system responsible for the attachment of ubiquitin to the target protein consists of several components that act in concert [3,6], including a ubiquitin-activating enzyme (E1), a ubiquitin-conjugating enzyme (E2), and a ubiquitin-protein isopeptide ligase (E3). E3 is thought to be the component of the ubiquitin conjugation system that is most directly responsible for substrate recognition. In addition, a new type of ubiquitylation enzyme, a ubiquitin chain assembly factor (E4), was recently discovered and shown to be required for the degradation of certain types of substrate, including an artificial fusion protein with an NH2-terminal ubiquitin moiety, via a ubiquitin fusion degradation (UFD) pathway [7,8]. Ufd2 of Saccharomyces cerevisiae is the prototype E4 enzyme. Ufd2 contains a conserved U-box domain, which appears to be an essential functional domain for E4 activity [9,10], and is associated with Cdc48 [8], which belongs to the large family of AAA-type ATPases that are thought to possess chaperone activity [11,12]. We have previously shown that mouse E4B (also known as UFD2a) is a homolog of yeast Ufd2, given that it contains a conserved U-box domain at its COOH-terminus and interacts with VCP, a mammalian ortholog of yeast Cdc48. These properties of E4B suggest that the association of AAA-type ATPases with Ufd2-like proteins that possess ubiquitylation activity has been conserved through evolution and may thus be functionally important [10,13].

The roles of E4B in vivo have remained largely unknown, however. E4B is expressed predominantly in neural tissues of adult mice [10], suggesting that it performs a neural-specific function. We found that E4B targets the pathological form of ataxin-3—in which abnormal expansion of a polyglutamine tract is responsible for spinocerebellar ataxia type 3 (SCA3) in humans—for ubiquitylation and degradation in mammalian cells as well as in a Drosophila melanogaster model of SCA3 [14]. Furthermore, we isolated FEZ1 (fasciculation and elongation protein zeta 1), a protein implicated in neurite extension, as a binding partner of E4B [15]. FEZ1 is a mammalian homolog of Caenorhabditis elegans UNC-76, which is required for axonal bundling and elongation in the nematode [16], suggesting that a FEZ1-E4B system also participates in axonal outgrowth and fasciculation in mammals. Other groups also reported that UFD2a is implicated in the process of Wallerian degeneration of neurons [17,18]. Moreover, we showed that E4B+/- mice manifest axonal dystrophy in the nucleus gracilis as well as degeneration of Purkinje cells associated with endoplasmic reticulum stress, and that these animals develop a neurological disorder [13]. Mice nullizygous for E4B died in utero as a result of developmental defects in the heart, suggesting an additional role for E4B in developmental processes in this organ. In spite of these various observations, however, the precise physiological functions of this enzyme remained elusive.

## Neurodegeneration and obesity in mice transgenic for E4B

**Figure 1.**
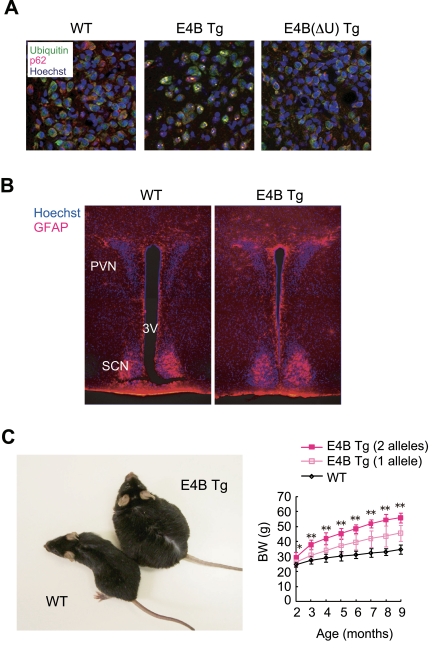
** E4B transgenic (Tg) mice as a new obesity model with hypothalamic neurodegeneration.** (**A**) Immunofluorescence analysis of the PVN region of 6-month-old wild-type (WT) or E4B(ΔU) Tg mice and of a 4-month-old E4B Tg mouse. Brain slices were stained with antibodies to polyubiquitin (green) and to p62 (red), and nuclei were stained with Hoechst 33258 (blue). Protein aggregates reacted with both types of antibody in the PVN region of E4B Tg mice, but not in that of WT or E4B(ΔU) Tg mice. (**B**) Immunofluorescence analysis of the PVN region of 10-week-old WT or E4B Tg mice with antibodies to glial fibrillary acidic protein (GFAP, red). Nuclei were stained with Hoechst 33258 (blue). SCN and 3V indicate the suprachiasmatic nucleus and third ventricle, respectively. The number of GFAP-positive glial cells in and around the PVN was increased in E4B Tg mice, indicative of gliosis associated with neurodegeneration. (**C**) Obesity in E4B Tg mice. The gross appearance of an E4B Tg mouse and a WT littermate at 9 months of age is shown on the left. The time course of body weight (BW) for WT mice and E4B Tg lines harboring one or two alleles of the transgene is shown on the right. The extent of obesity in the Tg animals harboring two alleles of the transgene was about twice that in littermates harboring only one allele, indicating that the obese phenotype is directly related to the expression level of the transgene. *P < 0.05, **P < 0.01 for the Tg line with two alleles of the transgene versus wild-type mice.

During further studies to explore the roles of E4B, we discovered that overexpression of E4B in a neural cell line resulted in the formation of protein aggregates that were recognized by antibodies to ubiquitin as well as by those to p62, a marker of ubiquitin-associated aggregates. This phenomenon was also reproduced in E4B transgenic (Tg) mouse lines in which expression of the E4B transgene is controlled by the promoter of the gene for the mammalian prion protein [19] (Figure 1A). This aggregate formation is apparently dependent on the ubiquitylation activity of the enzyme, given that few such aggregates were detected in cells expressing E4B(ΔU), a truncated form of E4B that lacks the catalytic U-box domain (Figure 1A). In addition, an important feature of the aggregates is that they resemble ubiquitin- and p62-positive aggregates observed in many human neurodegenerative diseases or in mice with neurodegeneration resulting from defects in autophagy, another pathway for the clearance of cellular components [20,21].

The aggregate formation in E4B Tg mice was apparent specifically in certain hypothalamic nuclei. Among these nuclei, the aggregate-associated neurodegeneration was most obvious in the paraventricular nucleus (PVN). PVN neurons are activated by signaling downstream of food intake [22-25], and they function as a satiety center. Indeed, lesions in the PVN result in the development of hyperphagic obesity in rat [26]. Furthermore, neurodegeneration-associated gliosis was observed in the region adjacent to the PVN in the hypothalamus of E4B Tg mice (Figure 1B), indicating that ectopic expression of E4B results in the formation of ubiquitin-positive aggregates and associated pathological features characteristic of neurodegenera-tive diseases [27].

Surprisingly, the E4B Tg mice were unequivocally obese (Figure 1C) and manifested increased lipid accumulation in tissues such as adipose tissue and the liver [27]. We investigated whether this obese phenotype was attributable to functional impairment ofthe hypothalamic satiety center. The animals exhibited increased food intake and decreased energy expenditure as well as several abnormal responses of the center to satiety input, indicating that malfunction of the hypothalamic satiety center is responsible for the obese phenotype of the E4B Tg mice. Finally, we observed that the Tg mice manifested metabolic disorders seen in obese humans.

On the basis of our observations, we proposed that the E4B Tg mouse is a new animal model for neurodegeneration-associated obesity that possesses several advantages. First, these animals spontaneously develop obesity and thus do not need to be fed a high-fat diet. Second, they manifest abnormalities in the highly restricted area of the hypothalamic satiety center and thus exhibit pathological features similar to those of some other mouse models of obesity, such as ob/ob and db/db mice, in which the hypothalamic leptin circuit is impaired [28,29]. Third, only one allele of the E4B transgene is required for mice to develop obesity. Furthermore, the extent of obesity can be varied by selection of transgenic lines with different levels of expression or different numbers of alleles of the transgene (Figure 1C), whereas most other mouse models are loss-of-function mutants and therefore require homozygosity of the mutant allele for manifestation of the phenotype. Fourth, E4B Tg mice also develop leptin and insulin resistance, glucose intolerance, hypercholesterolemia, and hypoadipo-nectinemia during progression of the obesity phenotype. These characteristics thus suggest that E4B Tg mice recapitulate the course of human obesity.

## Perspective

Our genetic mouse model has also provided the first experimental demonstration that neurodegeneration can indeed result in obesity, suggesting that some cases of human obesity might be attributable to hypothalamic neurodegeneration in aged individuals without any other neural disorders including cognitive and behavioral dysfunction. Aberrant activity of E4B might be a possible cause of obesity and associated metabolic disorders in humans, a notion that is consistent with the localization of obesity-related genetic markers in the vicinity of the E4B gene locus [30,31]. Further analysis of E4B function, particularly through identification of its substrates, should provide greater insight into the pathological properties of the molecule. More generally, nonspecific neurodegeneration associated with aging might result in a tendency to become obese. Together, our findings with E4B Tg mice open a new field of research linking obesity and aging processes as represented by degeneration of neural tissue.
